# 4D Micro-Computed X-ray Tomography as a Tool to Determine Critical Process and Product Information of Spin Freeze-Dried Unit Doses

**DOI:** 10.3390/pharmaceutics12050430

**Published:** 2020-05-07

**Authors:** Brecht Vanbillemont, Joris Lammens, Wannes Goethals, Chris Vervaet, Matthieu N. Boone, Thomas De Beer

**Affiliations:** 1 Laboratory of Pharmaceutical Process Analytical Technology (LPPAT), Department of Pharmaceutical Analysis, Ghent University, Ottergemsesteenweg 460, 9000 Ghent, Belgium; Brecht.Vanbillemont@UGent.be; 2 Laboratory of Pharmaceutical Technology, Department of Pharmaceutics, Ghent University, Ottergemsesteenweg 460, 9000 Ghent, Belgium; Joris.Lammens@UGent.be (J.L.); Chris.Vervaet@UGent.be (C.V.); 3 Radiation Physics Research Group, Department of Physics and Astronomy, Ghent University, Proeftuinstraat 86, 9000 Ghent, Belgium; Wannes.Goethals@UGent.be (W.G.); Matthieu.Boone@UGent.be (M.N.B.); 4 Centre for X-ray Tomography (UGCT), Ghent University, Proeftuinstraat 86, 9000 Ghent, Belgium

**Keywords:** freeze-drying, 4D-μCT imaging, process design, endpoint analysis, mechanistic modelling, process simulation, continuous processing

## Abstract

Maintaining chemical and physical stability of the product during freeze-drying is important but challenging. In addition, freeze-drying is typically associated with long process times. Therefore, mechanistic models have been developed to maximize drying efficiency without altering the chemical or physical stability of the product. Dried product mass transfer resistance ($R_{p}$) is a critical input for these mechanistic models. Currently available techniques to determine $R_{p}$ only provide an estimation of the mean $R_{p}$ and do not allow measuring and determining essential local (i.e., intra-vial) $R_{p}$ differences. In this study, we present an analytical method, based on four-dimensional micro-computed tomography (4D-μCT), which enables the possibility to determine intra-vial $R_{p}$ differences. Subsequently, these obtained $R_{p}$ values are used in a mechanistic model to predict the drying time distribution of a spin-frozen vial. Finally, this predicted primary drying time distribution is experimentally verified via thermal imaging during drying. It was further found during this study that 4D-μCT uniquely allows measuring and determining other essential freeze-drying process parameters such as the moving direction(s) of the sublimation front and frozen product layer thickness, which allows gaining accurate process knowledge. To conclude, the study reveals that the variation in the end of primary drying time of a single vial could be predicted accurately using 4D-μCT as similar results were found during the verification using thermal imaging.

## 1. Introduction

Freeze-drying or lyophilisation of pharmaceutical unit doses is a low temperature drying technique where water, or other solvents, are removed from temperature-sensitive products via sublimation [1]. Freeze-drying or lyophilisation consists of three consecutive steps: freezing, primary drying (i.e., sublimation) and secondary drying (i.e., desorption) [2]. Despite its popularity, freeze-drying is still an inefficient process associated with high production costs and long process times [3]. A reduction in production cost and process time can be achieved by shifting from batch freeze-drying to continuous freeze-drying. An innovative continuous freeze-drying process was recently developed [4,5,6,7,8]. The freezing step used in this continuous freeze-drying process is modified compared to traditional batch freeze-drying, as glass vials filled with a liquid formulation are rotated around their longitudinal axis while cooled and frozen with a cold, sterile and inert gas (i.e., spin freezing) [4,5,6,7,8]. This approach reduces the product layer thickness and increases the surface area, resulting in much higher sublimation rates and thus much shorter production times. A complementary method to decrease the production time involves using mechanistic models describing the drying step. A lot of research has been conducted last decades to develop mechanistic models for primary and secondary drying in order to maximise the drying rate, while maintaining the critical quality attributes (CQA) of the product [9,10,11,12,13]. The appearance of the cake (i.e., the layer of dried material) is an important CQA and is highly dependent on the collapse temperature of the product ($T_{c}$). Once the product temperature ($T_{p}$) exceeds $T_{c}$, molecular motion induces structural loss of the cake. Therefore, the $T_{p}$ should be kept below $T_{c}$ during primary drying [2,14].

The sublimation velocity depends on the local structure of the dried product. During sublimation, an ice-free layer is formed above the frozen product layer. Water molecules escaping from the sublimation front have to travel across this porous dried product matrix. The flow of these water molecules through this porous matrix is restricted by the size and tortuosity of the pores of this matrix, i.e., the dried product mass transfer resistance ($R_{p}$) [15,16]. A local rise in the partial water vapour pressure ($P_{i}$) occurs if the maximum mass flow through the pores is reached, leading to an increase in sublimation front temperature ($T_{i}$). Therefore, $R_{p}$ is one of the most important inputs for mechanistic primary drying models [13]. Furthermore, $R_{p}$ is highly dependent on the size of the ice crystals formed during freezing, and highly impacts the process cycle design [17,18]. Vial-to-vial and intra-vial variability in ice crystal size were recently reported, indicating that $R_{p}$ is not uniform over the batch and even over the entire vial [19,20]. However, currently available techniques for $R_{p}$ determination only provide an average $R_{p}$ at vial or batch level [21,22,23,24]. An analytical method to determine the $R_{p}$ distribution over the entire vial would be very useful in elucidating intra-vial variability of the drying characteristics.

Micro-CT is a powerful non-destructive microscopy technique that has become widely available in the last decade. Micro-CT allows non-destructive visualisation and analysis of a sample in three dimensions with a spatial resolution in the order of a few micrometers [25]. However, 3D characterization of the pore structure of a fully freeze-dried cake does not necessarily provide detailed information about the drying kinetics [26]. Usually, the 3D characterization of the pore structure is used to give an estimation of the $R_{p}$ via mass transfer modelling, which can be mathematically challenging [16]. Furthermore, 3D characterization usually requires static conditions to avoid image blurring and other motion artefacts. 4D-μCT or time-resolved μCT enables the possibility to image dynamic processes by capturing 3D-volumes in function of time, adding a temporal dimension. Recently, enormous improvements in the domain of 4D-μCT have been made. 4D-μCT enables the possibility of fast image acquisition, even on a sub-second time-scale [27,28,29]. By mapping differences between the 3D-volumes, dynamic parameters describing the process under investigation can be regressed. This allows the visualization of dynamic processes via μCT without the need to interrupt the process.

These $R_{p}$ distributions will provide detailed information about the cake structure which can be used during formulation and process development. Furthermore, the effect of local differences in pore structure will be better understood, allowing better controlled process conditions.For instance, the $R_{p}$ distribution and differences in the frozen layer thickness at start of primary drying are essential parameters in primary drying process simulations, such as the prediction of the primary drying endpoint distribution. An endpoint distribution could assist in the decision to proceed to secondary drying. Advancing to secondary drying when certain parts of the vials are not completely free of crystal water could lead to local meltback or collapse, impairing chemical and physical stability and other critical quality attributes of the freeze-dried cake [2,12].

It is the aim of this paper to evaluate the use of 4D-μCT in lyophilisation and in particular its ability to determine the $R_{p}$ vs. dried layer thickness ($L_{dr}$) profile per azimuth-height coordinate (i.e., a cylindrical angular-height segmentation) for a spin freeze-dried vial. This would lead to the establishment of a $R_{p}$ distribution over the entire cake. Furthermore, it will be investigated if 4D-μCT could reveal the drying kinetics such as sublimation rate by directly analysing the 3D-volumes without the need of mass transfer modelling. In addition, the possibility of 4D-μCT to display the impact of cake artefacts, such as cracks and shrinkage, on the lyophilisation process will be researched.

## 2. Materials and Methods

### 2.1. Materials

Two model formulations were used in this study, a 3% (w/V) mannitol solution and a 4% (w/V) bovine serum albumin (BSA) solution in deionised water. Both were selected because of their differences in solid state, i.e., mainly crystalline for the dried mannitol formulation and amorphous for the BSA formulation. Furthermore, both formulations have a high $T_{c}$ (−2 ${}^{\circ }$C for mannitol and −9 ${}^{\circ }$C for BSA), minimizing the risk of (micro-)collapse during the time-resolved μCT experiments. All materials were purchased from Sigma Aldrich (Zwijnaarde, Belgium).

### 2.2. Determination of Collapse Temperature

The collapse temperature ($T_{c}$) of the model formulations was determined via freeze-dry microscopy. A FDCS 196 freeze-drying stage (Linkam, Surrey, UK) was mounted underneath an optical microscope (BX51, Olympus, Hamburg, Germany). A sample of 3 μL was placed on top of a quartz sample window. Next, a thin metal U-shaped spacer was placed around the sample. Subsequently, the sample and spacer were covered with a glass coverslip. Finally, the freeze-drying chamber was closed with a hermetical lid.

The sample was frozen by lowering the temperature of the freeze-drying stage to −45 ${}^{\circ }$C via the Linksys32 software (Linksys 32, Linkam, Surrey, UK). The pressure of the sample chamber was decreased via a rotary vane vacuum pump to 1 Pa (E2M1.5, Edwards, Nazareth, Belgium) once the freezing-stage reached a temperature of −45 ${}^{\circ }$C. Next, the temperature of the stage was increased with a heating rate of 1.0 ${}^{\circ }$C/min. Meanwhile, the structure of the dried product was monitored via a digital camera which was mounted on top of the microscope. $T_{c}$ was defined as the temperature at which fissures were visible in the freshly formed dried product layer. The experiment was executed three times for each formulation.

### 2.3. Spin-Freezing

**Figure 1:**
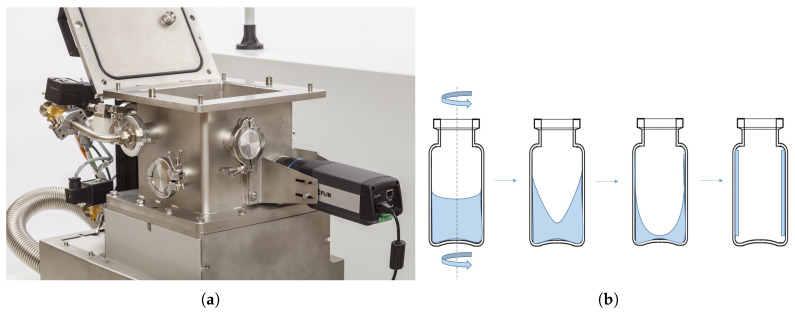
(**a**) Picture of a Rheavita spin-freezer with the thermal camera in front of a germanium window and (**b**) the graphical representation of the spin-freezing concept. Adapted with permission from Corver J., Eur. Pharm. Rev.; published by Russell Publishing Ltd., [30].

A 10R glass vial (Fiolax clear, Schott, Lukácsháza, Hungary) was filled with 3.5 mL formulation prior to spin-freezing. This filled glass vial was positioned vertically in the spin-freezer (Rheavita, Zwijnaarde, Belgium) depicted in Figure 1a [5]. Next, the vial was rotated at 2900 rpm around its longitudinal axis while jetting it with cold compressed air (130 L/min, −60 ${}^{\circ }$C) for 6 min as displayed in Figure 1b. Meanwhile, the temperature of the vial was monitored with a thermal infrared (IR) camera (FLIR A655sc, Thermal focus, Ravels, Belgium). This thermal IR camera was located in front of the spin-freezer, measuring the temperature of the glass vial through a germanium window. The temperature of the vial was monitored to assure that the temperature at the end of spin-freezing was well below −45 ${}^{\circ }$C. Finally, the vials were stored on dry ice (i.e., −78.5 ${}^{\circ }$C) for transport to the μCT scanner.

### 2.4. In-Situ Micro-CT Set-Up

#### 2.4.1. EMCT Scanner

**Figure 2:**
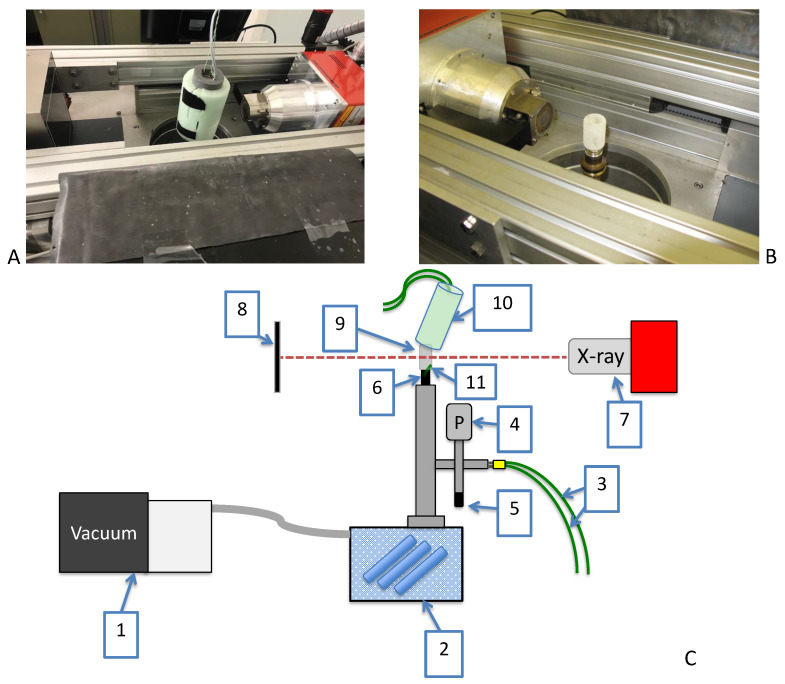
(**A**) μCT set-up with spin-frozen vial wrapped with insulating fabric surrounded by X-ray source and detector. (**B**) Spin-frozen vial without insulation (merely to illustrate the positioning of the vial). (**C**) 1: Vacuum pump; 2: Condenser; 3: Frozen product thermocouple read-out; 4: Proportional pressure valve; 5: Pirani gauge; 6: Vial manifold 7: X-ray source; 8: Detector; 9: Spin-frozen vial; 10: Heating pad wrapped with insulating fabric.; 11: thermocouple located inside vial at vial neck.

The μCT scanner used in this set-up is an in-house built high-resolution scanner developed for in-situ monitoring (EMCT scanner, UGCT, Belgium). This gantry based μCT scanner rotates horizontally around the sample and allows fast and continuous scanning of objects, particularly when peripheral equipment is used. The EMCT is equipped with a closed 130 kV X-ray source and a CMOS flat panel detector with 1316 by 1312 pixels [31]. The 10R vial was positioned in between the source and detector to have the full image of the frozen product inside the vial, resulting in a resolution of 30 μm (Figure 2).

#### 2.4.2. Freeze-Drying Set-Up

A custom-built single-vial manifold freeze-dryer was installed in the middle of the μCT scanner. (cfr. Figure 2). This custom-built freeze-dryer consisted of a condenser which was located underneath the μCT scanner. A rotational vacuum pump (DS102, Agilent technologies, Leini, Italy) was connected to the condenser to evacuate the system. The pressure of the system was monitored via a VD84 Pirani gauge (Thyracont vacuum instruments, Passau, Germany) and controlled via a EVR116 proportional pressure valve (Pfeiffer Vacuum, Asslar, Germany), both connected to the vial manifold.

A 10R vial filled with 3.5 mL of the formulation was spin-frozen as described in Section 2.3. The spin-frozen vial was pulled vacuum (and thus dried) by mounting it on the custom-built single-vial freeze-dryer, installed in the middle of the μCT scanner. Transfer from dry ice to the set-up was achieved in 10 s to limit heating of the vial. An electric heating pad was tightly wrapped around the spin-frozen vial to provide the heat required for sublimation. An in-house scripted LabVIEW 2017 (National Instruments, Austin, TX, USA) application was used to keep the electric heating pad around the spin frozen vial at a fixed temperature of 40 ${}^{\circ }$C and the pressure level at 5 Pa using proportional–integral–derivative (PID) controllers [4]. In addition, a low attenuating insulating fabric was put over the spin-frozen vial to shield it from uncontrolled energy from the environment.

Furthermore, the temperature of the ice layer was monitored via a thin gauge type-K thermocouple (Labfacility, Leeds, United Kingdom) located at the ice surface near the vial neck. In addition, two extra thermocouples were installed, one at the bottom of the spin-frozen vial and one at the top of the heating pad to monitor the temperature of the spin-frozen vial and heating pad, respectively. The thickness of the frozen product layer was monitored during primary drying via a μCT scanner. Therefore, the scanner rotated constantly around the spin-frozen vial while recording. A full vial scan was made each 176.11 s during primary drying, recording 1600 projections per rotation. The process was imaged during 40 (BSA) or 35 (mannitol) sequential rotations in total (i.e., 117 and 103 min of primary drying, respectively). From this data, 40 (BSA) and 69 (mannitol) 3D volumes were reconstructed at a time interval of 2.93 min (BSA) and 1.47 min (mannitol).

#### 2.4.3. Data Structure

**Figure 3:**
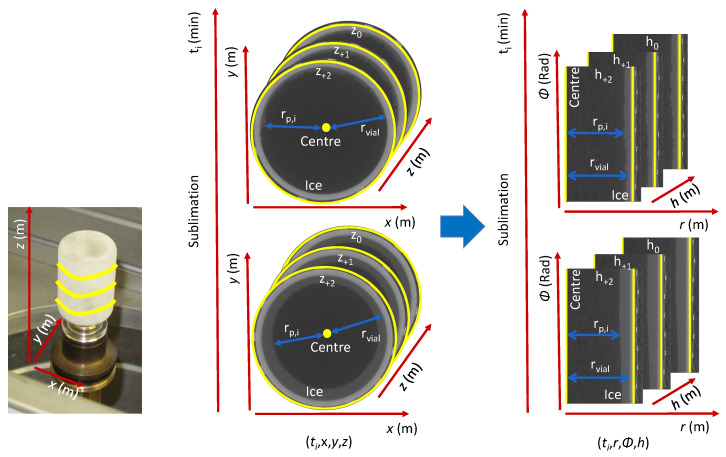
Conversion of the data structure from a cartesian coordinate system (x,y,z) (middle section) to a cylindrical coordinate system (*r*, Φ, *h*) (right section) for each time point $t_{i}$, (i.e., each scan).

Tomographic reconstruction of all scans was performed using the in-house CTrex software (UGCT, Ghent, Belgium). For all 3D volumes, i.e., each time point ($t_{i}$), the data-structure was converted from a cartesian coordinate system (*x*, *y*, *z*), with 1300 segmentations in each direction at a voxel size of (30 μm)${}^{3}$, to a cylindrical coordinate system (*r*, Φ, *h*) with 650 radial (*r*), 1600 azimuthal (Φ) and 1300 height (*h*) segmentations which resulted in a 4D matrix ($t_{i}$, *r*, Φ, *h*) as represented in Figure 3. The amount of x-rays absorbed by an object is defined by Beer’s law as a function of the attenuation coefficient and thickness of a material. The dried product layer formed during freeze-drying has a negligibly low attenuation coefficient in comparison with the dense frozen product and glass layer. Therefore, only the frozen product layer could be segmented from the vial-assembly by thresholding the greyscale value per voxel. At last, for all ($t_{i}$, Φ, *h*) coordinates the inner radius of the frozen product layer ($r_{p}$) and inner vial wall ($r_{vial}$) was calculated.

### 2.5. Direction of Sublimation

Two algorithms were developed to evaluate if, for all ($t_{i}$, Φ, *h*) coordinates, the direction of sublimation was primarily radial (*r*) (i.e., from centre of the vial to vial wall) and if sublimation front irregularities were present. At first, the thickness of the frozen product layer ($L_{ice}$) was calculated by the difference between the radial coordinates of the inner vial wall ($r_{vial}$) and of the frozen product ($r_{p}$) closest to the centre of the vial ($L_{ice,i}=r_{vial}-r_{p,i}$). Secondly, an integrated frozen layer length ($L_{ice}^{\prime }$) was computed by summation of the length of all voxels in the radial direction that were categorised as ice. Cavities in the frozen layer would not be covered by the $L_{ice}^{\prime }$ parameter as these are not be classified as ice. Conversely, they are included in the $L_{ice}$ parameter as they are located within $r_{vial}$ and $r_{p,i}$. Significant difference between $L_{ice}$ and $L_{ice}^{\prime }$ would indicate the existence of these cavities which can only originate from multiple sublimation interfaces in the radial direction and would be a sign of considerable non-radial sublimation. Lastly, for every time point ($t_{i}$) a surface plot was constructed to display both the $L_{ice}$ and the relative difference ($\left(L_{ice}-L_{ice}^{\prime }\right)/L_{ice}$) for all azimuthal and height coordinates.

### 2.6. Determination of Dried Product Mass Transfer Resistance

$R_{p}$ (m/s) describes the relationship between the driving pressure difference ($P_{i}$ – $P_{c}$) and the surface area normalized sublimation rate (Equation (1)) [1]. The dry layer thickness ($L_{dr}$) versus $R_{p}$ profiles were determined for every ($t_{i}$, Φ, *h*) coordinate in the data matrix using the following equations.

(1) $$R_{p}=\frac{A_{p}\left(P_{i}-P_{c}\right)}{{\dot{m}}_{sub}}$$

where $A_{p}$ is the projected product area ($m^{2}$), $P_{i}$ the vapour pressure of ice at the sublimation front (Pa), $P_{c}$ the partial pressure in the drying chamber (Pa) and ${\dot{m}}_{sub}$ the sublimation rate (kg/s). $P_{i}$ was determined via Equation (2) which is an empirical equation based on the Clausius-Clapeyron relation between the sublimation front temperature ($T_{i}$) (K) and vapour pressure of ice with $\alpha_{P_{i}}$ till $\delta_{P_{i}}$ empirical coefficients [32].

(2) $$P_{i}=e^{\alpha_{P_{i}}-\frac{\beta_{P_{i}}}{T_{i}}+\gamma_{P_{i}}\ln T_{i}-\delta_{P_{i}}T_{i}}$$

The thickness of the frozen product layer ($L_{ice,i}$) was extracted from the data structure at each time point (i.e., 3D volume) for all (Φ, *h*) coordinates. A Savitzky-Golay smoothing (third degree polynomial and window of 15) of $L_{ice}$ over the time was applied to increase the precision of the data without distorting the signal tendency. Subsequently, considering the cylindrical geometry of the cake (Figure 3), the volume $V_{i}$ of frozen product within each azimuthal-height segmentation was calculated in function of time ($t_{i}$) according Equation (3).

(3) $$V_{i}=\frac{\pi h_{vial}}{n\cdot m}\left(r_{vial}^{2}-r_{p,i}^{2}\right)$$

where, $h_{vial}$ is the height of the vial (m), n the number of azimuthal segmentations of the vial (-) and m the number of height segmentations of the vial (-).

Subsequently, the sublimation rate ${\dot{m}}_{sub}$ could be calculated according to Equation (4) from the change in frozen product volume per time point ($\frac{dV_{i}}{dt}$) (m${}^{3}$/s) by taking the dried layer porosity ($\theta_{dr}$) (-) and ice density ($\rho_{ice}$) (kg/m${}^{3}$) into account.

(4) $${\dot{m}}_{sub,i}=\frac{dV_{i}}{dt}\rho_{ice}\theta_{dr}$$

As a one-dimensional freeze-drying model was assumed, the frozen product area ($A_{p,i}$) available for sublimation (m${}^{2}$) was calculated according to Equation (5) by considering the projected surface area of the part of the voxel oriented to the centre of the vial. Hence, the main sublimation direction was considered to be radial. Any minor freeze-drying phenomena leading to a diversion from the main radial direction was still included in the model as it was regressed into the formulation and (micro-)structure parameter $R_{p}$.

(5) $$A_{p,i}=\frac{2\pi }{n\cdot m}h_{vial}\left(r_{p,i}\right)$$

$R_{p}$ can be expressed in function of time or in function of dry layer thickness ($L_{dr}$). The dry layer thickness was calculated per azimuthal and height coordinate according to Equation (6). Note that not necessarily every (Φ, *h*) coordinate reaches the same dry layer thickness at an identical point in time.

(6) $$L_{dr,i}=L_{ice,0}-L_{ice,i}$$

where $L_{dr,i}$ is the dried layer thickness (m) for a specific time point, $L_{ice,0}$ is the initial ($t_{0}$) ice layer thickness (m) and $L_{ice,i}$ the ice layer thickness at a certain scan ($t_{i}$) (m).

The $R_{p}$ was calculated using Equations (1) till (6) with the frozen product temperature measured by thermocouple near the vial necks as the sublimation temperature ($T_{i}$). Next, surface plots with the (Φ, *h*) coordinate values of $L_{dr}$, ${\dot{m}}_{sub}$ and $R_{p}$ were constructed for every time point related to a 3D-volume. Finally, the $R_{p}$ data was also plotted as a function of $L_{dr}$ by binning the latter into 38 bins ranging from 0 to 2.5 mm to facilitate the graphical representation of the $R_{p}$ at similar $L_{dr}$ instead of time.

### 2.7. Simulation of the Primary Drying Time Distribution

The $R_{p}$ profiles of BSA determined in the previous sections were used to simulate the primary drying time for every azimuthal and height coordinate of the vial (Φ, *h*). A similar continuous process for BSA was modelled but with a chamber pressure ($P_{c}$) of 10 Pa and a total power of 1.2 W ($P_{tot}$) towards the vial. However, to decrease the computational time of this simulation, spatial binning over the azimuthal (10×) and height (10×) dimensions were necessary, resulting in 100 height and 160 azimuthal coordinates per time point.

To estimate the heat balance of the whole vial system, the median $L_{dr,vial}$ vs. $R_{p,vial}$ profile was determined from the BSA dataset calculated in the previous section. At each time iteration of the prediction (Δt: 60 s), the sublimation interface temperature ($T_{i}$) of the whole vial system was estimated by simultaneously solving Equations (2), (7) and (8) with $A_{p,vial}$ the sublimating surface of the whole system (m${}^{2}$), *M* the molecular weight of water (kg/mol), $\Delta H_{sub}$ the latent heat of sublimation (J/mol) and $\alpha_{H_{sub}}$ till $\epsilon_{H_{sub}}$ the empirical coefficients describing the $\Delta H_{sub}$ in function of the temperature [12].

(7) $$P_{tot}=\frac{A_{p,vial}\left(P_{i}-P_{c}\right)}{R_{p,vial}}\cdot \frac{\Delta H_{sub}}{M}$$

(8) $$\Delta H_{sub}=\alpha_{H_{sub}}+\beta_{H_{sub}}T_{i}-\gamma_{H_{sub}}T_{i}^{2}+\delta_{H_{sub}}e^{-{\left(\frac{T_{i}}{\epsilon_{H_{sub}}}\right)}^{2}}$$

Subsequently, the following actions were performed per (Φ, *h*) coordinate. At first, the dried layer resistance was estimated using a spline interpolation of the $L_{dr}$ vs $R_{p}$ profile of that coordinate calculated in Section 2.6. This was followed by a product surface ($A_{p,i}$) calculation according Equation (5). Next, ${\dot{m}}_{sub,i}$ could be computed by applying Equations (1) and (2). Furthermore, the sublimated volume ($V_{i}$) was calculated.

(9) $$V_{i}=\sum_{t_{0}}^{t_{i}}\frac{{\dot{m}}_{sub,i}\Delta t}{\theta \rho_{ice}}$$

With these parameters, the $L_{dr,i}$ of all ($t_{i}$, Φ, *h*) coordinates was approximated according to Equation (10) and compared to the initial frozen product thickness ($L_{ice,0}$) of every coordinate to check if sublimation was finished ($t_{end}$) for that specific (Φ, *h*) coordinate.

(10) $$L_{dr,i}=r_{vial}-L_{ice,0}-\sqrt{{\left(r_{vial}-L_{ice,0}\right)}^{2}-\frac{V_{i}}{\pi h_{vial}}}$$

At the end of every time iteration, a new heat balance of the vial was made using Equations (2), (7) and (8) to yield a new global $T_{i}$. With the $A_{p,vial}$ being estimated by summation of all radial facing sublimating voxel surfaces. $L_{dr,vial}$ was calculated as the mean of $L_{dr,i}$ from all coordinates still sublimating and used in the $L_{dr,vial}$ vs $R_{p,vial}$ spline interpolation to yield $R_{p,vial}$. Finally, a primary drying time ($t_{end}$) surface plot for all (Φ, *h*) coordinates was constructed. All parameter values used in the regression and simulation are listed in Table 1.

### 2.8. Verification Experiment

The primary drying time distribution predicted as described in Section 2.7 was verified via thermal imaging of an actual primary drying phase. A 10R vial filled with 3.5 mL BSA formulation was spin-frozen according to Section 2.3. Subsequently, the vial was placed into a single-vial drying-chamber (Rheavita, Zwijnaarde, Belgium), where the spin-frozen vial rotated at 5 rpm in front of an infrared heater (Weiss Technik, Zellik, Belgium). Next, a rotary vacuum pump was switched on to lower the chamber pressure and thus inducing primary drying. As soon as the chamber pressure reached a pressure level of 10 Pa, the infrared heater was switched on (total power towards vial = 1.2 W, calculated according to the method described in [5]). Meanwhile, the temperature of the rotating vial was recorded with a thermal camera (FLIR A655sc, Thermal focus, Ravels, Belgium). The frame rate of the thermal camera was synchronised with the rotation velocity of the drying vial. In addition, the horizontal field of view of the thermal camera was calculated according to Equation (11), allowing the construction of a temperature distribution over the entire vial through time.

(11) $$HFOV=2d_{w}tan\left(\frac{\alpha }{2}\right)$$

where HFOV is the horizontal field of view of the thermal camera (m), $d_{w}$ is the distance between the thermal camera and the rotating vial (m) and α is the field of view angle (${}^{\circ }$). A sharp increase in the mean temperature of the vial indicated that primary drying has come to an end [5]. The total primary drying time for each pixel was determined by verifying when this temperature raise occurred for each pixel of the vial wall. Finally, an additional primary drying time distribution was hence obtained via these thermal measurements. The primary drying time distribution achieved via μCT was compared to the time distribution obtained via thermal imaging.

## 3. Results and Discussion

### 3.1. Direction of Sublimation

**Figure 4:**
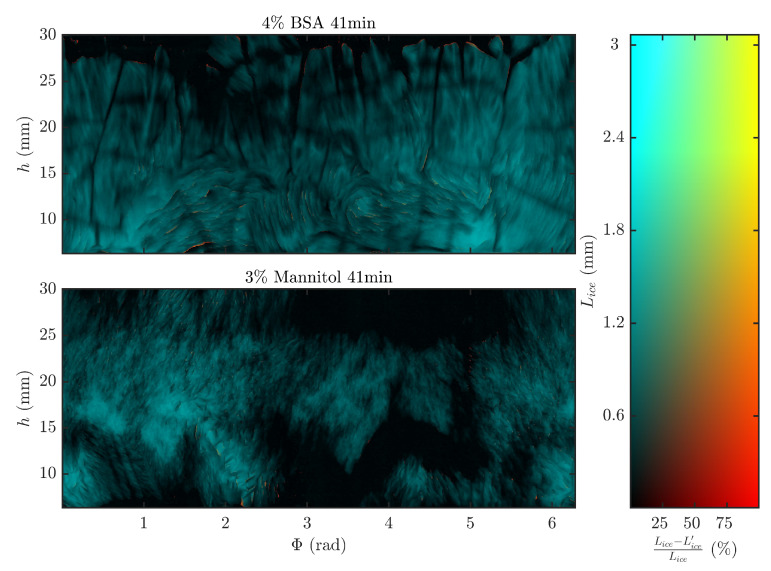
Graphical representation of the relative difference of the frozen product thickness ($L_{ice}$) and integrated frozen product length ($L_{ice}^{\prime }$) for every (Φ, *h*) coordinate by means of a 2D-colour scale (right) for 4% BSA (top left) and 3% mannitol (bottom left) at 41 min.

Surface plots similar to Figure 4 were constructed for all the 3D volumes of BSA and mannitol. A surface colour in between black and cyan (i.e., left side of the colour scale) is indicative for a $L_{ice}^{\prime }$ that is as large as $L_{ice}$. In case $L_{ice}^{\prime }$ is smaller than $L_{ice}$, the coordinate will appear orange to yellow and indicates the existence of cavities inside the frozen product layer. Furthermore, the brighter the coordinate appears, the higher its $L_{ice}^{\prime }$ and $L_{ice}$ are. As can be perceived on Figure 4, no to very minimal orange is measured, which was also the case for all other time points in the BSA and mannitol samples. Situations where $L_{ice}^{\prime }$ < $L_{ice}$ can only originate if non-radial sublimation occurs, creating cavities in the ice layer. Such cavities result in multiple sublimation fronts in the radial direction of one (Φ, *h*) coordinate. This was only observed to a significant extent during preliminary tests of the setup with sublimation of deionised water without excipients. Since sublimation was mainly noticeable in the radial direction in the case of mannitol or BSA formulations, it is very likely that the dried product layer of spin freeze-dried samples prevents this complex non-radial behaviour. However, local differences in the progress of primary drying were still noticeable between the (Φ, *h*) coordinates as different grades of cyan and black are simultaneously visible in Figure 4. It stresses the importance of intra-vial ${\dot{m}}_{sub}$ and $R_{p}$ calculations. A one-dimensional freeze-drying model describing a sublimation front moving from the centre of the vial towards the vial wall was assumed since the moving direction of the sublimation front was mainly radial. Therefore, only the surface of the voxels facing the centre of the vial were presumed to sublimate in the $R_{p}$ estimation. Consequently, all other freeze-drying behaviour, i.e., crack formation and slight non-radial sublimation resulting in irregular topology is covered by the formulation and structure parameter $R_{p}$.

### 3.2. Frozen Product Thickness and Sublimation Rate

**Figure 5:**
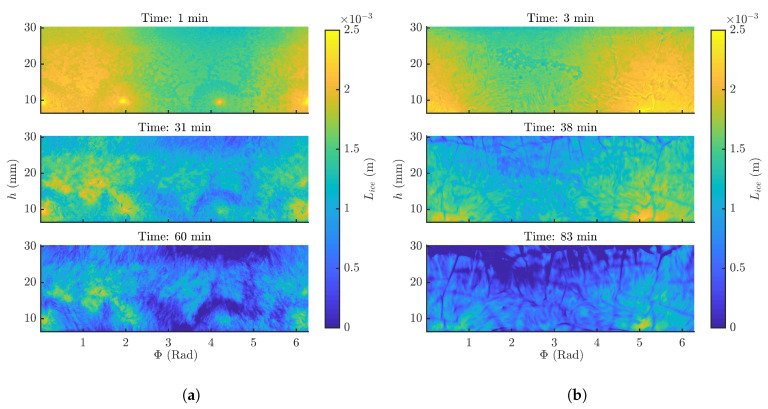
(**a**) Frozen product layer thickness $L_{ice,i}$ for every (Φ, *h*) coordinate in function of time of a 3% mannitol formulation during spin freeze-drying (**b**) Frozen product layer thickness $L_{ice,i}$ for every (Φ, *h*) coordinate in function of time of a 4% BSA formulation during spin freeze-drying.

The decrease in frozen product layer thickness over time is visualised in Figure 5a,b for mannitol and BSA formulations, respectively. During spin-freezing the liquid formulation is spread over the entire vial wall. If the rotation velocity of the vial during spin-freezing is not high enough, a difference in frozen product layer thickness between top and bottom of the vial is expected [6]. The first measurement point (3 min for mannitol and 1 minute for BSA) revealed that this was the case for both formulations. A difference of up to respectively 15% and 23% in initial average frozen product layer thickness ($L_{ice,1}$) between top and bottom of the vial was observed. These differences in initial layer thickness were expected (i.e., 20%) and earlier described by Lammens et al. [6]. Rotational velocities of more than 5700 rpm would be necessary to limit layer thickness differences in height to less then 5%. In addition, the first measurement point revealed azimuthal differences up to 1 mm in frozen product layer thickness over the vial at one height level. Minor vial geometry defects or a slight misalignment of the vial in the spin-interface introduced azimuthal differences in frozen product layer thickness, since the vial was spinned outside its true centre. Differences in layer thickness can induce intra-vial differences in drying time. However, these differences in drying time can be completely controlled during production, since the entire surface of all vials are monitored via thermal imaging (cfr. Section 3.5). The vial slowly rotates in front of the thermal camera having the whole vial in scope. Moreover, the primary drying end point of the complete vial can be determined very accurately as demonstrated by Van Bockstal et al. [5].

Furthermore, global differences in frozen product layer structure could be observed between both formulations. As visible in Figure 5b, some cracks in the frozen product layer of the BSA formulation were present during sublimation. These cracks were not clearly visible in the first measurement point, possibly because their size was still below the measurement resolution. The cracks are interruptions in the frozen product layer which can induce differences in drying kinetics as explained in following sections. However, these cracks were not present in the frozen product layer of mannitol. These differences might be a consequence of the differences in solid state since mannitol is mainly crystalline, while BSA is mainly amorphous. Moreover, it should be noted that cracks can be avoided by optimizing the bulk formulation composition [14]. Shrinkage and cracking are a result from relaxing the drying tension built up by water removal. There is an inverse correlation between shrinkage and cracking. If shrinkage is restrained due to the mechanical strength of the cake then more cracking occurs to relax the drying tension. The mechanical strength of the cake is related to the solid state of the product and influences therefore the occurrence of cracks [33].

On the other hand, the frozen product layer of mannitol exhibits some remarkable circular spots at the bottom of the vial (cfr. Figure 5a). These very local differences in frozen product layer thickness correspond with the position of the grippers used to hold the vial in vertical position during spin-freezing. These grippers covered a part of the vial during spin-freezing and prevented direct contact between the cold gas and the vial, therefore a local difference in frozen product layer thickness might be induced. Similar ice expansion phenomena are regularly seen at the centre top of a batch freeze-dried cake. As these are the last locations where liquid is present during freezing, all the ice expansion is directed toward these spots. It is clear that an optimisation of the gripper design is advisable to mitigate the azimuthal differences by misalignment and to counter the freezing artefacts at the location of the grippers. By minimizing the gripper adaptors and changing the material into a high conductive fabric would most probably lead to less interference. Moreover, redirecting more cooling power to the bottom of the vial which was also associated with higher frozen layer thicknesses could be another suggested optimisation.

**Figure 6:**
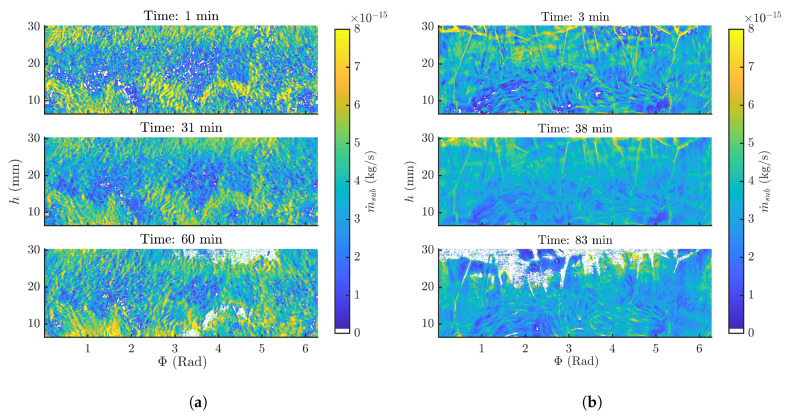
(**a**) Sublimation rate (${\dot{m}}_{sub}$) for every (Φ, *h*) coordinate in function of time of a 3% mannitol formulation during spin freeze-drying. (**b**) Sublimation rate (${\dot{m}}_{sub}$) per (Φ, *h*) coordinate in function of time of a 4% BSA formulation during spin freeze-drying.

The sublimation rate was derived from the decrease in ice layer thickness for every (Φ, *h*) coordinate as described in Equation (4). The sublimation rate distributions at multiple time points of the mannitol and BSA formulation are represented in Figure 6a,b, respectively. As visualised in these figures, local differences in sublimation rate across the entire vial were present, revealing a possible distribution in $R_{p}$. These local differences were observed for both formulations, indicating that sublimation rate distributions over the vial might be present regardless of the solid state of the dried product.

Evaluating the sublimation rate over time revealed that at some locations, the sublimation rate increases, as visible in Figure 6. An increase in sublimation rate is usually not expected, since as drying proceeds a longer $L_{dr}$ is formed which should be accompanied with a longer and more restricting Knudsen flow [23]. The ${\dot{m}}_{sub}$ increase can only be attributed to a unusual decrease in $R_{p}$. Micro-structural changes during processing could possibly explain such behaviour.

### 3.3. Dry Product Resistance Distribution

**Figure 7:**
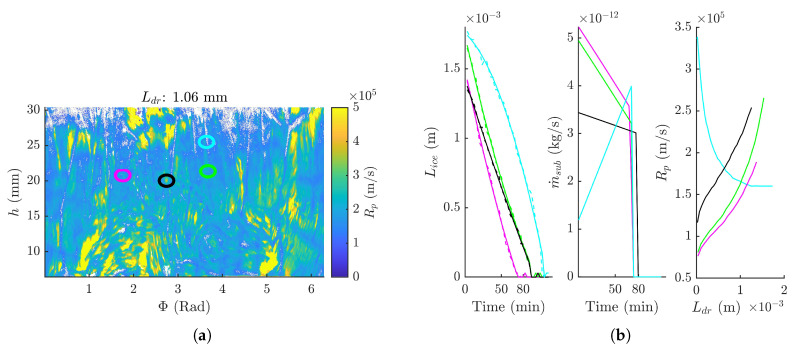
(**a**) $R_{p}$ at a $L_{dr}$ of 1.06 mm for every (Φ, *h*) coordinate for the 4% BSA formulation. Four noteworthy different regions (circles) were selected in the surface plot. (**b**) Following data were plotted for each of the selected point: $L_{ice}$ in function of time (left) with dashed lines the raw data and full lines the smoothed data; ${\dot{m}}_{sub}$ in function of time (middle); $R_{p}$ in function of $L_{dr}$ (right).

$R_{p}$ in function of $L_{dr}$ for every (Φ, *h*) coordinate was calculated as described in Section 2.6. The $R_{p}$ distribution of the BSA formulation at a $L_{dr}$ of 1.06 mm is represented in Figure 7. As can be deduced from Figure 7a, differences in $R_{p}$ are present across the vial. Some noteworthy regions were highlighted in Figure 7b. For instance, the $R_{p}$ profiles of regions in the immediate vicinity of a crack strongly differs from a theoretical textbook $R_{p}$ profile (Region highlighted in cyan in Figure 7), as it decreased over time. This is correlated with an increase in sublimation rate over time (cfr. Figure 7b), most likely caused by the formation of the crack, since this interrupts the dried layer, thus facilitating the transport of water molecules through the dried layer.

Although the shape of the $R_{p}$ profiles of the other selected points in Figure 7a are very similar, some $R_{p}$ profiles are considerably higher than others. A slightly higher sublimation front temperature might be expected in regions with a higher $R_{p}$, therefore increasing the risk of (micro-) collapse. In addition, differences in $R_{p}$ across the vial will induce differences in primary drying time across the vial as explained in Section 3.4. These locally different $R_{p}$ profiles are most likely caused by differences in pore morphology. For instance, regions with a higher $R_{p}$ might correlate with a locally higher tortuosity or a smaller pore size. These possible intra-vial structural differences will be investigated in future work. Local differences in pore morphology are inherent to freeze-drying as they partly originates from local heat flux differences at ice nucleation and consolidation during the freezing phase [34]. These heat flux differences are generally much higher in batch freeze-drying, so we would also expect a broader $R_{p}$ distribution. However, they can be minimized by a more uniform heat transfer (i.e., continuous processing) or by controlled nucleation techniques in the case of batch processing [18,19].

### 3.4. Primary Drying Time Distribution

**Figure 8:**
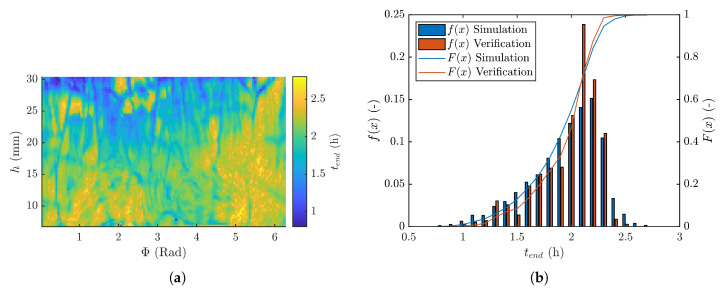
(**a**) Distribution of primary drying endpoint ($t_{end}$) for a simulation of a spin-freeze dried 10R vial with 3.5 ml 4% BSA formulation at a chamber pressure of 10 Pa and total power towards the vial of 1.2 W and (**b**) End of primary drying ($t_{end}$) histograms (f(x)—left axis) and cumulative distribution (F(x)—right axis) for both the simulation (Section 3.4) and the verification (Section 3.5).

From the $R_{p}$ and $L_{ice,0}$ distribution of the BSA formulation, a prediction of the primary drying time ($t_{end}$) for every (Φ, *h*) coordinate was made and is depicted in Figure 8a. Clear differences in $t_{end}$ are noticeable across the vial, which varies between 1 and 2.7 h. The $t_{end}$ results are regionally grouped with generally higher $t_{end}$ towards the bottom of the vial. However, there is also significant azimuthal asymmetry. This coincided with the results of the initial frozen product thickness of BSA (cfr. Figure 5b), evidenced by a correlation coefficient of 0.5292 between $t_{end}$ and $L_{ice,1}$ with a 95% confidence interval (CI) from 0.5169 and 0.5419. It is evident that the product thickness inhomogeneity has a major influence on the process design, especially on the primary drying endpoint.

Another significant contributor to $t_{end}$ is $R_{p}$. Upon comparing the sublimation rate of BSA (Figure 6b) with the primary drying time (Figure 8a), considerable regional similarities can be recognized, particularly when comparing the areas with cracks. The higher sublimation rate was caused by a lower product resistance ($R_{p}$) and reduced primary drying times. This is evidenced by a correlation coefficient between the initial product resistance $R_{p,0}$ (at $L_{ice,0}$) and $t_{end}$ of 0.3255, with a 95% CI from 0.3095 till 0.3413. Macro-structural phenomena, such as cracking, or micro-structural morphology differences are inducing significant local $R_{p}$ differences, impacting the primary drying time.

### 3.5. Verification Experiment

As explained in Section 3.4, differences in $R_{p}$ and initial frozen product layer thickness across the vial induce noteworthy intra-vial variability in primary drying time. This highlights the importance of an accurate in-process analytical tool to determine $t_{end}$. Thermal imaging has proven to be a very valuable tool to determine $t_{end}$ accurately [5].

Thermal imaging was used as a tool to verify the $t_{end}$ distributions obtained via μCT methodology. The distribution in primary drying time across the vial acquired via thermal imaging is represented in Figure 8b. A quite similar distribution was obtained using thermal imaging compared to the μCT based primary drying simulation. The total predicted drying time is quite comparable with the total drying time of the verification experiment. In addition, the global trend in both distributions is very similar. Therefore, it was concluded that thermal imaging was successfully used as a process analytical tool to determine intra-vial differences in $t_{end}$. This enables the possibility to use real-time feedback control to proceed to secondary drying and avoiding the risks of (micro-)collapse. Nonetheless, the described 4D μ-CT technique enables the possibility to determine intra-vial differences with a higher resolution than via thermal imaging. In addition, thermal imaging can only be used to detect intra-vial differences in drying time, the physical origin of the variation would be difficult to elucidate using thermal imaging. In contrast, 4D μ-CT generates 3D-volumes in function of time which can aid in the discovering the origin of the phenomena (i.e., cracking, freezing artefacts ..) leading to drying time variation. Moreover, freeze-drying is a drying technique based on the principles of heat and mass balance. Thermal imaging is a process analytical technology based on the heat balance while 4D μ-CT is a mass balance technique, which makes them both very complementary.

## 4. Conclusions and Future Perspectives

The in-situ μCT methodology was capable of recording a freeze-drying process and directly distilling essential process parameters such as the frozen product layer or sublimation rate. Furthermore, a framework is proposed to measure the local frozen product differences and to calculate their respective local $R_{p}$ profiles. Moreover, the impact of the distribution of the initial frozen product layer thickness and product mass transfer resistance on the primary drying time was identified to be significant. It can be concluded that specific attention should be given to intra-vial inhomogeneity during freeze-cycle design, especially the decision to switch to secondary drying. Prematurely starting secondary drying could have a major impact on the product appearance and product stability. However, it was also established that in-process thermal imaging of the vial during primary drying is a justifiable alternative, as the primary drying endpoint distribution was comparable to the μCT methodology. In future work, the origin of the discovered differences in $R_{p}$ will be further explored via high resolution μCT scans of the dried layer. Furthermore, a similar investigation should be executed on traditional batch freeze-drying. A broader $R_{p}$ distribution would be expected in batch processing as generally a longer and more tortuous restrictive Knudsen flow will be present.

## Figures and Tables

**Table 1:** Parameters and variables list. A dash as value represents a variable.

Description	Symbol	Value	Unit
Field of view angle	α	45.1	${}^{\circ }$
Latent heat of sublimation	$\Delta H_{sub}$	-	J/mol
Time resolution prediction	Δt	60	s
Dried layer porosity	θ	0.97	(-)
Mass density ice	$\rho_{ice}$	918	kg/m${}^{3}$
Azimuth (cylindrical coordinate)	Φ	-	Rad
$P_{i}$ coefficient	$\alpha_{Pi}$	9.550426	Pa
$P_{i}$ coefficient	$\beta_{Pi}$	5723.2658	K
$P_{i}$ coefficient	$\gamma_{Pi}$	3.53068	1/K
$P_{i}$ coefficient	$\delta_{Pi}$	0.00728332	Pa
$\Delta H_{sub}$ coefficient	$\alpha_{H_{sub}}$	4.68 × 10${}^{4}$	J/mol
$\Delta H_{sub}$ coefficient	$\beta_{H_{sub}}$	35.9	J/molK
$\Delta H_{sub}$ coefficient	$\gamma_{H_{sub}}$	0.0741	J/molK${}^{2}$
$\Delta H_{sub}$ coefficient	$\delta_{H_{sub}}$	542	J/mol
$\Delta H_{sub}$ coefficient	$\epsilon_{H_{sub}}$	124	K${}^{2}$
Product surface	$A_{p}$	-	m${}^{2}$
Product surface entire vial	$A_{p,vial}$	-	m${}^{2}$
Distance camera - vial	$d_{w}$	$15\times 10^{-2}$	m
Height (cylindrical coordinate)	*h*	-	m
Height of vial	$h_{vial}$	$32\times 10^{-3}$	m
Horizontal field of view	HFOV	$12\times 10^{-2}$	m
Dried product thickness	$L_{dr}$	-	m
Frozen product thickness	$L_{ice}$	-	m
Initial frozen product thickness	$L_{ice,0}$	-	m
Integrated frozen product length	$L_{ice}^{\prime }$	-	m
Sublimation rate	${\dot{m}}_{sub}$	-	kg/s
Molecular weight water	*M*	$18.01528\times 10^{-3}$	kg/mol
Height segmentations (Section 2.6/Section 2.7)	*m*	1000/100	(-)
Azimuthal segmentations (Section 2.6/Section 2.7)	*n*	1600/160	(-)
Chamber Pressure (Section 2.6/Section 2.7 and Section 2.8)	$P_{c}$	5/10	Pa
Partial water vapour Pressure	$P_{i}$	-	Pa
Total power towards vial	$P_{tot}$	1.2	W
Radius (cylindrical coordinate)	*r*	-	m
Radius frozen product	$r_{p}$	-	m
Radius inner vial wall	$r_{vial}$	$10.9\times 10^{-3}$	m
Dried layer resistance	$R_{p}$	-	m/s
Time point	$t_{i}$	-	min
Primary drying time	$t_{end}$	-	h
Collapse temperature (BSA/mannitol)	$T_{c}$	−9/−2	${}^{\circ }$C
Sublimation interface temperature	$T_{i}$	-	K
Product temperature	$T_{p}$	-	K
Volume cylindrical segment	*V*	-	m${}^{3}$
Length (cartesian coordinate)	*x*	-	m
Width (cartesian coordinate)	*y*	-	m
Depth (cartesian coordinate)	*z*	-	m
